# Circular RNA cTFRC acts as the sponge of MicroRNA-107 to promote bladder carcinoma progression

**DOI:** 10.1186/s12943-019-0951-0

**Published:** 2019-02-19

**Authors:** Hongwei Su, Tao Tao, Zhao Yang, Xing Kang, Xu Zhang, Danyue Kang, Song Wu, Chong Li

**Affiliations:** 1 Department of urology, Affiliated Traditional Chinese Medicine Hospital, Southwest Medical University, Luzhou, 646699 China; 2 Core Facility for Protein Research, Institute of Biophysics, Chinese Academy of Sciences, Beijing, 100101 China; 3 Department of Urology, The Affiliated Luohu Hospital of Shenzhen University, Shenzhen University, Shenzhen, 518000 China; 4 College of Life Science and Technology, Beijing University of Chemical Technology, Beijing, China; 5 Beijing Jianlan Institute of Medicine, Beijing, 100190 China; 6 Michigan State University, 426 Auditorium Rd, East Lansing, MI 48824 USA; 7 Department of Urology, Urology Institute of Shenzhen University, The Third Affiliated Hospital of Shenzhen University, Shenzhen University, Shenzhen, 518000 China; 8 Department of Clinical Medicine, School of Medicine, Anhui University of Science and Technology, Huainan, 232001 China; 9 Department of Urology, Minimally Invasive Surgery Center, The First Affiliated Hospital of Guangzhou Medical University, Guangzhou, 510000 China; 10 Beijing Zhongke Jianlan Biotechnology Co., Ltd., Beijing, 101400 China

**Keywords:** Bladder Cancer, cTFRC, miR-107, TFRC, Circular RNA

## Abstract

**Background:**

Circular RNA (circRNA) represents a broad and diverse endogenous RNAs that can regulate gene expression in cancer. However, the regulation and function of bladder cancer (BC) circRNAs remain largely unknown.

**Methods:**

Here we generated circRNA microarray data from three BC tissues and paired non-cancerous matched tissues, and detected circular RNA-cTFRC up-regulated and correlated with tumor grade and poor survival rate of BC patients. We subsequently performed functional analyses in cell lines and an animal model to support clinical findings. Mechanistically, we demonstrated that cTFRC could directly bind to miR-107 and relieve suppression for target TFRC expression.

**Results:**

We detected circular RNA-cTFRC up-regulated and correlated with tumor grade and poor survival rate of BC patients. Knock down of cTFRC inhibited invasion and proliferation of BC cell lines in vitro and tumor growth in vivo. Furthermore, the expression of cTFRC correlated with TFRC and negatively correlated with miR-107 both in BC cell lines and BC clinical samples. In addition, up-regulation of cTFRC promoted TFRC expression and contributed to an epithelial to mesenchymal transition phenotype in BC cells. Finally, we found that cTFRC acts as a competing endogenous RNA (ceRNA) for miR-107 to regulate TFRC expression.

**Conclusions:**

cTFRC may exert regulatory functions in BC and may be a potential marker of BC diagnosis or progression.

**Electronic supplementary material:**

The online version of this article (10.1186/s12943-019-0951-0) contains supplementary material, which is available to authorized users.

## Background

Bladder cancer (BC) ranked the 9th most common cancer in the world, with a significant morbidity and mortality [1]. According to the Global Cancer Statistics, about 79,030 new cases of bladder cancer are diagnosed annually in the United States, and an estimated 16,870 patients will die of this disease [2]. While the most of first diagnosed bladder cancers present as noninvasive early tumors, up to one-third of non-muscle invasive bladder cancer (NMIBC) will progress to muscle invasive bladder cancer (MIBC) and metastasize to other organs over time [3], which highlights the urgent need for novel biomarkers and pathways to more accurately predict bladder cancer recurrence and cancer treatment.

The existence of circRNAs was first observed in eukaryotic cells nearly 40 years ago by using an electron microscope [4]. Initially, circRNA was occasionally reported and misinterpreted as a by-product of aberrant RNA splicing or splicing errors [5, 6]. With the advent of high-throughput sequencing, thousands of circRNAs have been successfully identified in different cell lines and species [7]. However, little is known about their potential function and biogenesis process. Recently, circRNAs have been verified to be associated with several diseases such as brain dis-function or neurodegenerative diseases like Alzheimer’s disease and several cancers. Unlike linear RNAs, circRNAs have the prominent feature of non-canonical splicing with no free 3′ and 5′ end, which enables them to be resistant to RNA exonucleases [8, 9]. These observations suggest that circRNA may be a novel potential biomarker and therapeutic target for cancer. However, the elucidation of deregulated circRNAs and the identification of their functions remain an ongoing process in cancer investigation.

The dysregulation and function of microRNAs (miRNAs) have been extensively studied in almost every biological process. However, the expression profile and function of newly identified circRNAs in specific biological activities still need further investigation. Pandolfi et al., reported that RNAs can co-regulate each other as ceRNAs through competitively shared miRNAs [10]. Transcripts such as mRNAs, lncRNAs and pseudogenes can function as natural miRNA sponges by competitive binding with miRNA response elements (MREs) to inhibit their expression and function [11]. lncRNAs acting as ceRNAs have been confirmed by several studies, while circRNAs containing multiple MREs can also serve as highly effective miRNA sponges that regulate gene expression at the transcriptional or post-transcriptional level [12, 13]. The expression of circRNA is strictly regulated in different environments and the study of circRNA is still in its beginning. However, the role of circRNA in bladder cancer has not been fully elucidated.

In our study, we analyzed the expression profile of circRNAs in BC tissues and determined that circRNA cTFRC was significantly up-regulated in BC tissues and closely related to the prognosis of BC patients. We found that cTFRC may function as the sponge of miR-107 to up-regulate the expression of TFRC (transferrin receptor) and consequently promote BC progression. Therefore, cTFRC can serve as a biomarker for prognosis predication and as a potential therapeutic target for BC patients.

## Methods

### Human tissues and cell lines

Primary human BC and paired adjacent normal bladder tissues were obtained from patients during operation. With the guidance of a skillful pathologist, we collected normal bladder urothelium samples with a distance of ≥3 cm from the edge of the bladder cancer tissue. After surgical resection, all specimens were immediately frozen in liquid nitrogen. All human studies were reviewed and approved by the IRB of Institute of Biophysics, Chinese Academy of Sciences, and written informed consent was provided according to the World Medical Association Declaration of Helsinki. Clinicopathological classification and staging were determined according to the American Joint Committee on Cancer Classification Criteria. RNA expression profiles and matching clinical data on 433 bladder cancer patients were downloaded from the TCGA data portal (https://tcga-data.nci.nih.gov/). Bladder cancer cell lines EJ, T24, 5637, UMUC3, BIU87, J82, SW780 and bladder normal epithelial cell HCV29 were cultured in RPMI 1640 medium (Gibco, NY, USA) supplemented with 10% fetal bovine serum (FBS) (Gibco), 100 U/mL penicillin (Gibco) and 100 μg/mL streptomycin (Gibco). All the cells were incubated at 37 °C in a humidified atmosphere containing 5% CO_2_.

### Expression profile analysis of circRNAs

The circRNAs chip (Arraystar Human circRNAs chip; ArrayStar, Rockville, MD, USA), containing 5396 probes specific for human circRNAs splicing sites, was used. After hybridization and washing with samples, three pairs of BC samples (tumor tissues and matched nontumor tissues) were analyzed on the circRNAs chips. Exogenous RNAs developed by the External RNA Controls Consortium were used as controls.

### Real-time qRT-PCR analysis

Total RNA was extracted using TRIzol reagent (Invitrogen, Carlsbad, CA, USA). RNA concentration was measured by Nonodrop, and each paired sample was adjusted to the same concentration. Real-time qPCR was performed as described [14]. Glyceraldehyde 3-phosphate dehydrogenase (GAPDH) and U6 were used as the internal control. Primers sequences for the detected genes were listed in Additional file 1: Table S1.

### Plasmids construction and stable tansfection

Short hairpin RNA (shRNA) of cTFRC were synthesized by GenePharma (Shanghai, China), shcTFRC targeting to the junction region of the cTFRC sequence. The shRNA plasmid of TFRC was purchased from Santa Cruz (Dallas, USA). EJ and T24 cells were transfected with cTFRC and TFRC shRNAs plasmids using Lipofecamine 2000 (Invitrogen, Carlsbad, CA, USA). The sequences of the effective shRNAs were provided in Additional file 1: Table S2. The full-length cTFRC cDNA was cloned into pCDH-CMV-MCS-EF1-GFP + Puro (Geneseed Biotech, Guangzhou, China) to obtain the pCDH-cTFRC overexpression of cTFRC. Production of lentiviral particles and transduction of BC cells was performed as described [14].

### Biotin-labeled pull-down assay

Biotinylated cTFRC and miR-107 (GenePharma, Shanghai, China) pull-down assay with target mRNAs was performed as described earlier [15]. In brief, 1 × 10^7^ bladder cancer cells were harvested, lysed, and sonicated. The probe was incubated with probes-M280 streptavidin dynabeads (Invitrogen) at 25 °C for 2 h to generate probe-coated beads. The cell lysates were incubated with the probe-coated beads mixture at 4 °C overnight. After washing with the wash buffer, the RNA complexes bound to the beads were eluted and extracted with Trizol Reagent (Invitrogen) for the analysis.

### Western blots

Cell lysates were prepared with RIPA buffer (Thermo Scientific). Immunoreactive bands were detected by using the Immobilon ECL substrate kit (Millipore, Merck KGaA, Germany). Antibodies used included primary antibodies against TFRC (Cat. No: ab218544, 1:1000 dilution, Abcam, USA), E-cadherin (Cat. No: ab1416, 1:1000 dilution, Abcam, USA) and β-actin (Cat. No: ab8226 1:1000 dilution, Abcam,); HRP-conjugated secondary goat anti-mouse (Cat. No: SA00001–1) or goat anti-rabbit (Cat. No: SA00001–2) antibodies (1:4000 dilution, Proteintech, USA).

### Cell invasion assay

The invasion assays were performed in 24-well FluoroBlok cell culture inserts (BD Biosciences) with 8-μm pore-size PET membrane. The insert was coated with 100 μL of 1 μg/μL Matrigel matrix (BD Biosciences) at 4 °C overnight. Following starvation for 6 h in serum-free RPMI 1640, cells were harvested from one subconfluent 10-cm dish by cell dissociation buffer (Life Technologies), spun at 500×g for 3 min, and resuspended in RPMI 1640. Cells (4 × 10^4^) in 500 μL of RPMI 1640 were seeded onto the insert and 750 μL of RPMI 1640 with 10% (vol/vol) FBS was added into the lower chamber of the transwells. After incubation for 18 h at 37 °C, the medium inside the insert was removed and the insert was then placed in a new 24-well plate. The invaded cells at the reverse side of the insert were labeled with a fluorescent dye Calcein AM (4 μM in Dulbecco’s PBS) (BD Biosciences) for 1 h at 37 °C. The fluorescence was measured with 494 nm/517 nm (excitation/emission wavelength) by a SpectraMax M5 microplate reader (Molecular Devices).

### [^3^H] thymidine incorporation

Cells were planted in 96-well plates and grown for 24 h after they were serum starved for 48 h. They were treated with 15d-PGJ_2_ for 48 h and pulsed with 5 μCi of [^3^H] thymidine for 4 h. We counted the radioactivity in Beckman L5 counter after washing the cells and stopping the reaction with 5% trichloroacetic acid and solubilizing the cells in 0.5% of 0.25 N sodium hydroxide. Each experiment was done in quadruplicates and repeated at least three times.

### Prediction of miRNA targets

CircRNA-miRNA interaction was predicted with miRNA target prediction software (Arraystar’s home-made) based on TargetScan and miRanda. TargetScan (http://www.targetscan.org) or miRBase (http://www.mirbase.org) were used to identify the miRNA targeting sites in TFRC 3’-UTR.

### Luciferase reporter assay

For luciferase reporter assay, pmirGLO Dual-luciferase vectors (GenePharma, Shanghai, China) were used to construct dual luciferase reporter plasmids. EJ or T24 cells were co-transfected with corresponding plasmids and microRNA, luciferase activity was assessed using the dual-luciferase reporter kit (Promega, Madison, WI, USA). The relative firefly luciferase activity was normalized to Renilla luciferase activity.

### Mice model

All animal studies were permitted by the Institutional Animal Care and Use Committee of the Institute of Biophysics, Chinese Academy of Sciences and were conducted in compliance with its recommendations. The stably knockdown of cTFRC EJ cells (2 × 10^6^) or T24 cells (2 × 10^6^) were subcutaneously injected into the back of 4-week-old male BALB/c mice. The tumor volume was monitored every 5 days. After 30 days, the mice were sacrificed and tumor tissues were excised and subjected to pathologic examination.

### Fluorescence in situ hybridization

Hybridization was performed overnight with cTFRC and miR-107 probes. Specimens were analyzed on a Nikon inverted fluorescence microscope. The cTFRC and miR-107 probe for fluorescence in situ hybridization (FISH) were listed in Additional file 1: Table S3.

### Statistical analysis

The Student t test was performed to analyze whether two experimental groups have significant difference using *P* < 0.05 as the significant criteria. Survival analysis was performed by Kaplan-Meier curves and log-rank test for significance in GraphPad Prism 5.

## Results

### cTFRC expression is significantly correlated with poor prognosis of BC patients

**Fig. 1:**
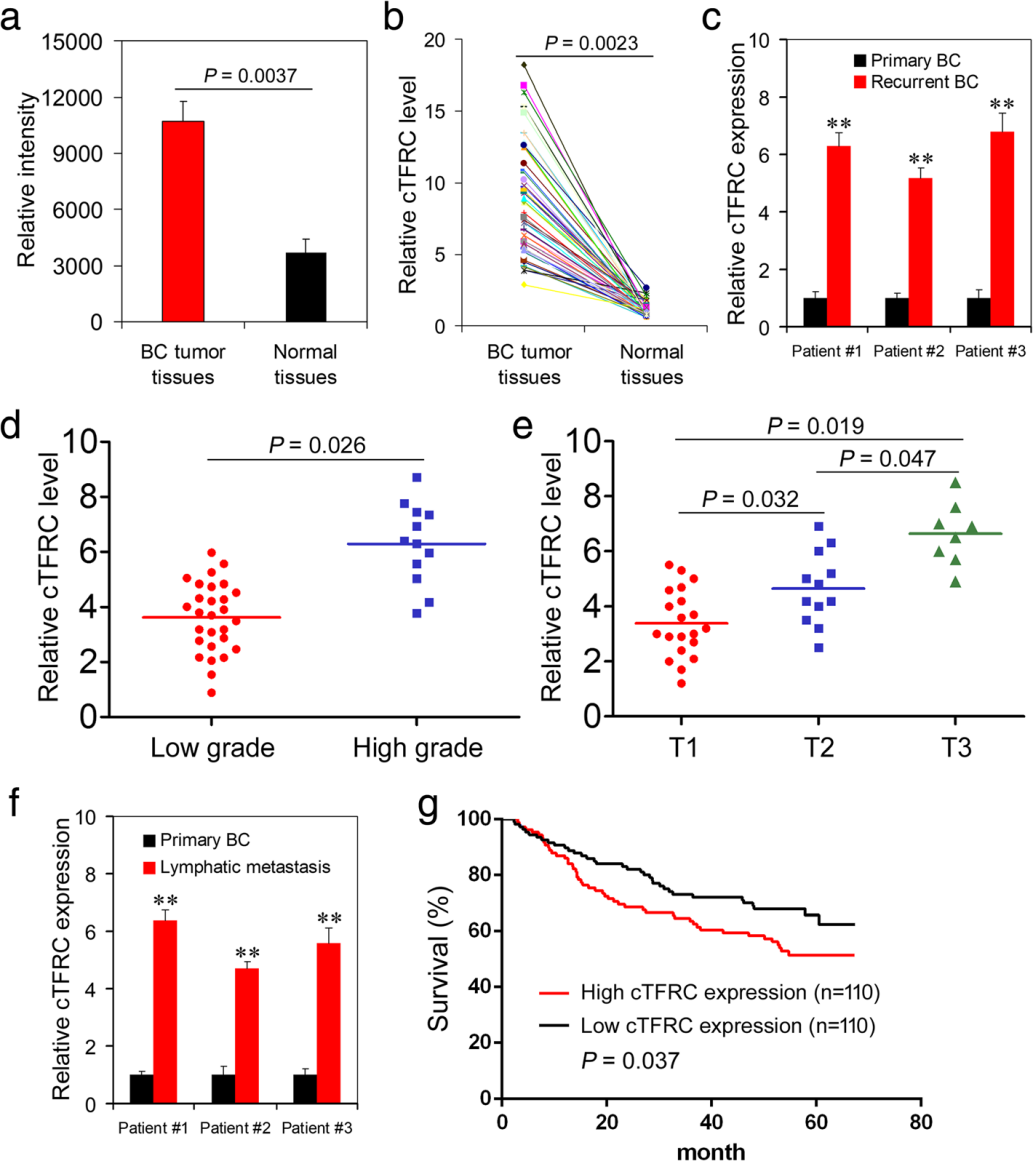
Up-regulated circRNAs in BC tumor tissues and its correlation with prognosis of patients. **a** cTFRC increased in BC tissues as compared to that in the matched nontumor tissues analyzed by circRNAs Arraystar Chip. **b** Schematic representation of the high expression level of cTFRC in 57 BC patients tissues compared with adjacent normal patients tissues by qPCR. **c** cTFRC upregulated in recurrent BC patients. **d** The high expression levels of cTFRC in BC patients with high grade. **e** Advanced T stage is associated with higher cTFRC levels. **f** The expression of cTFRC higher in patients with lymphatic metastasis. **g** Prognostic significance of cTFRC expression for BC patients was performed with cTFRC values by using the median value as the cutoff

To investigate the circRNA expression profile in BC tissues, we first analyzed three pairs of BC tissue samples (3 BC tissues and three matched non-tumor bladder tissues) by using the circRNA microarray. Through expression intensity sorting within BC tumor and non-tumor groups, we found that cTFRC expression was consistently and significantly increased in BC tumor tissues as compared to the matching controls (Fig. 1a). Therefore, we focused on the expression and role of cTFRC in BC progression in this study. To verify the microarray results, the expression of cTFRC in BC tissues was then confirmed by quantitative RT-PCR (qPCR). The 57 tumor samples had a higher expression level of cTFRC compared with their respective adjacent normal tissues (Fig. 1b). Because cTFRC expression was significantly increased in BC tissues, we then analyzed whether the increase in cTFRC expression was correlated with the prognosis of BC patients. A cohort of 220 BC patients with recurrence data were included, and cTFRC expression was determined using qPCR analysis. cTFRC expression was significantly increased in BC recurrent tissues as compared to that in the primary BC tissues (Fig. 1c). More importantly, the increased cTFRC expression in BC tissues was significantly correlated with higher grade and T stage in BC patients (Fig. 1d, e). Furthermore, the correlation analysis showed that cTFRC expression was up-regulated in BC patients with lymphatic metastasis (Fig. 1f). Kaplan-Meier’s survival curves showed that the patients with BC and higher cTFRC expression had poorer overall survival (Fig. 1g). The relationship between cTFRC expression and clinical characteristics of the BC patients were listed in Table 1. Taken together, these results suggested that the up-regulation of cTFRC was correlated with poor prognosis in BC patients.

**Table 1:** Relationship between the expression levels of cTFRC and clinicopathological features of baldder cancers

			Expression of aberrantly cTFRC		
Total		Patients	High	Low	*P*
Age					
Mean		51.6	52.3	50.9	0.635
Sex					
Male		174	102	72	0.518
Female		46	21	25	
Tumor stage					
T1		106	23	83	<0.001
T2		60	24	36	
T3		33	22	11	
T4		21	18	3	
Grade					
G1 or 2		161	89	72	<0.001
G3		59	47	12	
Number of tumors					
Solitary		183	97	86	0.057
Multiple		37	24	13	
Lymphatic invasion					
Negative		96	43	53	<0.005
Positive		72	52	20	
Unknown		52	23	29	
Follow-up (month)					
Mean		66.7	67.8	65.6	0.753

### Down-regulation of cTFRC inhibits invasion of BC cells

**Fig. 2:**
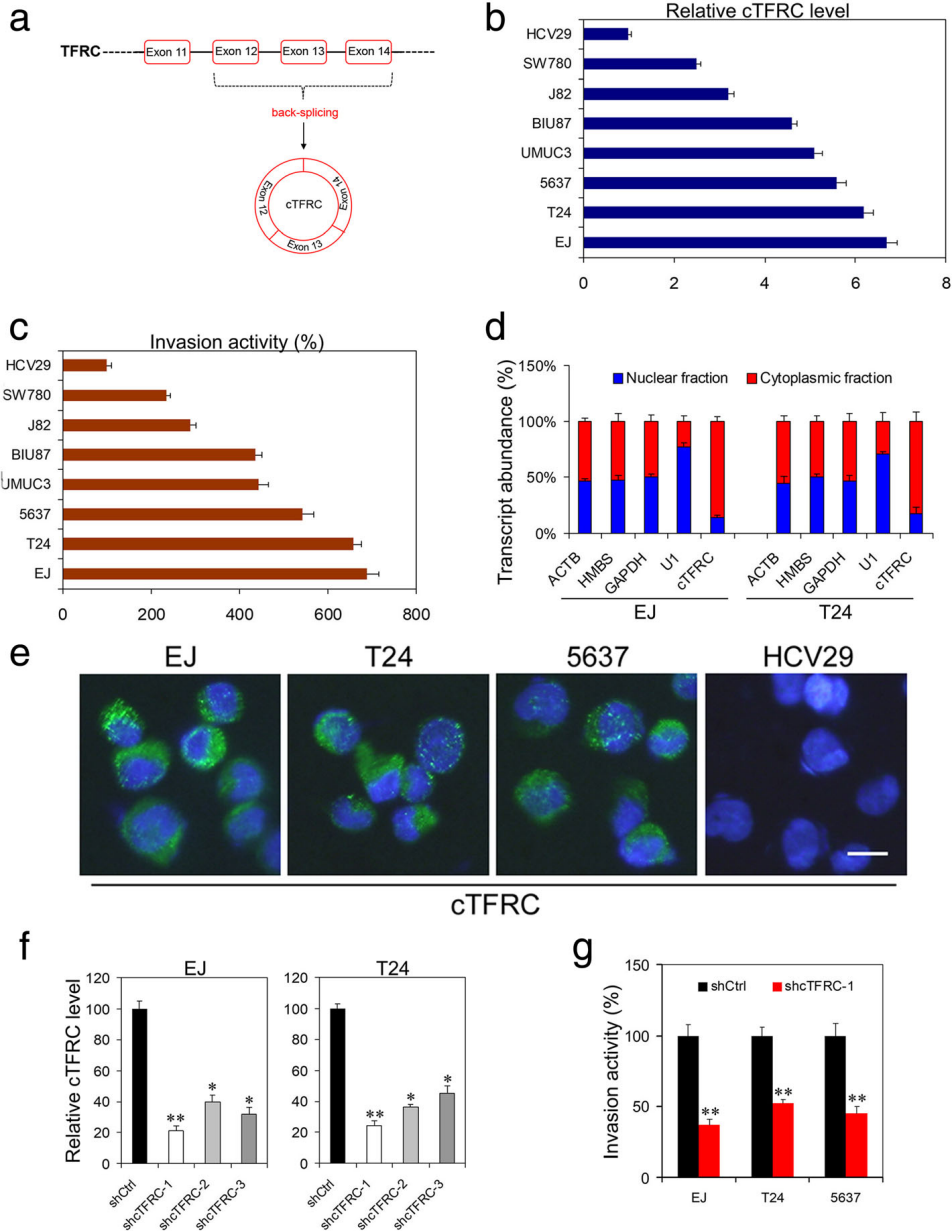
The effects of cTFRC on BC cell invasion. **a** Scheme illustrating the production of cTFRC. **b** The relative cTFRC level in BC cells. **c** Transwell assay different BC cells invasion activity. **d** Comparison of the abundance of cTFRC in the nuclear and cytoplasmic. Fractionation of EJ and T24 cells followed by qPCR. U1 RNA served as a positive control for nuclear gene expression. **e** RNA FISH for cTFRC. cTFRC probes were labeled with Cy3. Nuclei were stained with DAPI. Scale bar, 10 μm. **f** qPCR analysis of the transfection efficiency of shcTFRC vectors after transfection in EJ or T24 cells. **g** Determination of cell invasive potential of EJ, T24 or 5637 cells transfected with shcTFRC by Transwell assay. Data are the means ± SD of three independent experiments. *P < 0.05; **P < 0.01

We chose the circular RNA derived from exons 12, 13 and 14 of the TFRC gene [CircBase [16] ID: has-circ-0001445, termed cTFRC] and its precise genomic location is chr3:195785154–195,787,118 (Fig. 2a). We then explored the role of cTFRC in BC progression. To choose the BC cell lines used for silencing or overexpression of cTFRC, we first checked the expression of cTFRC in 8 BC cell lines (Fig. 2b). Meanwhile, we investigated the invasive ability of BC cell lines and found that cTFRC expression was positively correlated with cell invasiveness (Fig. 2c). EJ and T24 cells showed the high expression of cTFRC and the higher invasive activity. Therefore, EJ and T24 cells were selected for silencing cTFRC. We further investigated the abundance of the cTFRC. As shown in Fig. 2d and Fig. 2e, cTFRC was principally distributed in the cytoplasmic fraction. To analyze the role of cTFRC in cell invasion ability, we constructed three shRNAs which cover the back-splicing region of cTFRC to knock down the expression level of cTFRC. After transfection with cTFRC shRNAs into EJ and T24 BC cells, the efficiency of cTFRC silencing was measured and confirmed by qPCR (Fig. 2f). Silencing of cTFRC was performed in BC cells to determine its effect on BC progression. cTFRC silencing significantly reduced cell invasion of EJ, T24 and 5637 cells respectively (Fig. 2g). These findings indicated that under-expression of cTFRC could inhibit invasion of bladder cancer cells.

### Characterization of cTFRC in BC

**Fig. 3:**
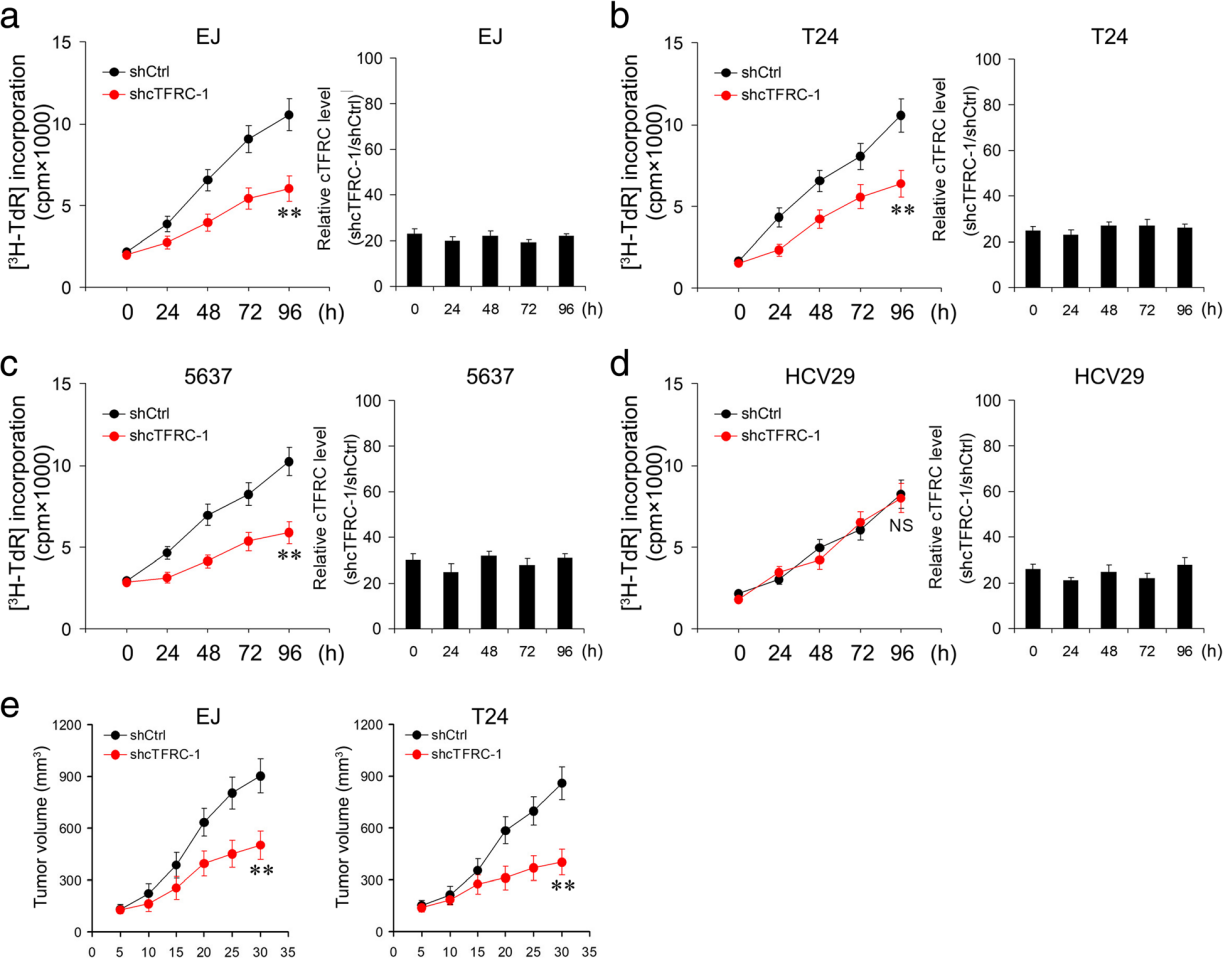
Silencing of cTFRC RNA inhibits BC cell proliferation. **a-d** Proliferation of EJ, T24, 5637 and HCV29 cells transfected with the above cTFRC shRNA-1 assessed using ^3^H-TdR incorporation at the indicated days. Left panel: Representative the growth curve of EJ, T24, 5637 and HCV29 cells transfected with shcTFRC-1. Right panel: Quantification of the expression of cTFRC at different time. Data in **a**-**d** are the mean ± SD of three experiments. **e** The growth curve of subcutaneous xenograft tumor from EJ or T24 cells in nude mice. shCtrl or shcTFRC-1 cells were subcutaneously injected into nude mice. Tumor size was measured every 5 days. Values were presented as the mean ± SD of 12 mice in each group. (Repeated-measures analysis of variance, **P < 0.01)

**Fig. 4:**
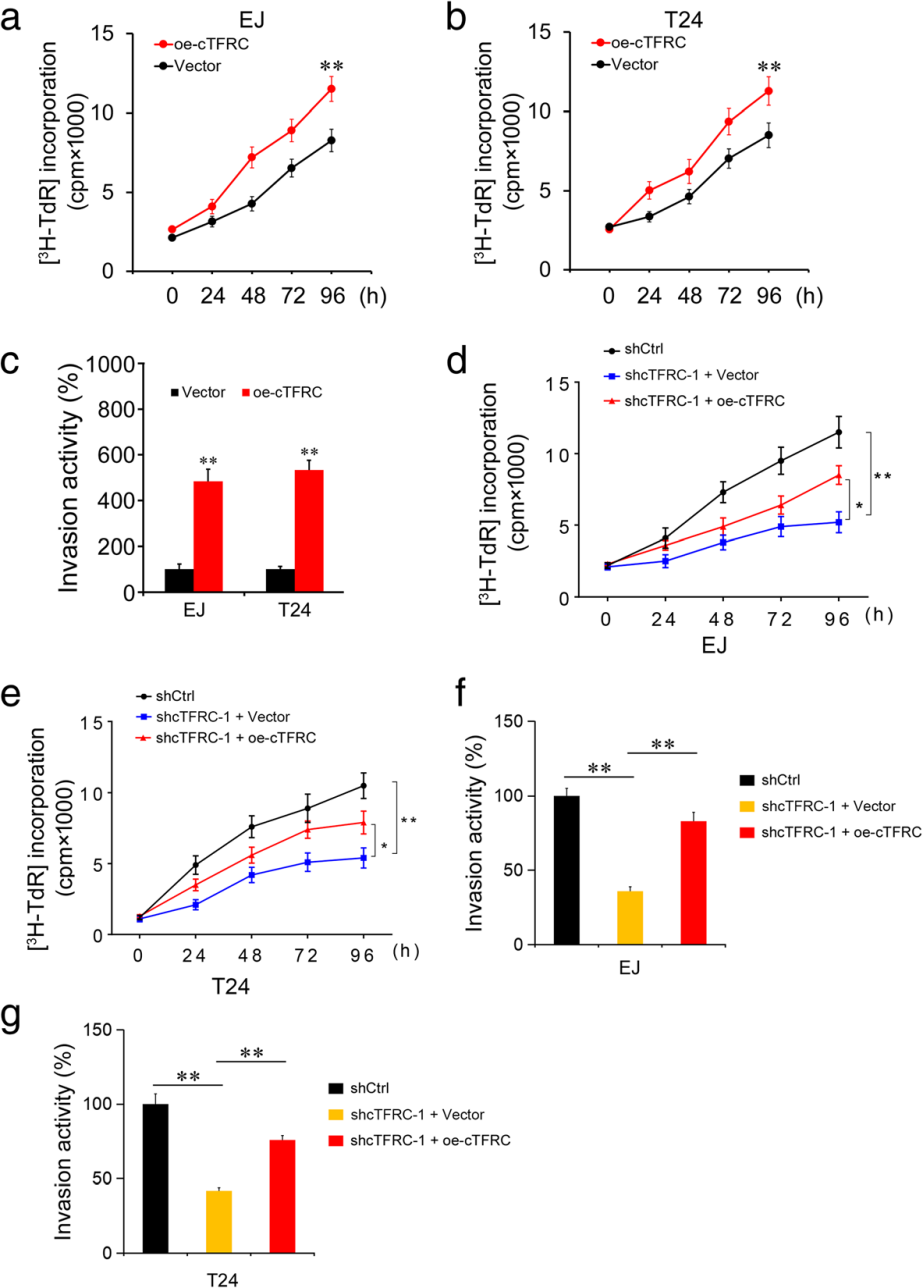
Overexpression of cTFRC induces BC cell proliferation and invasion. **a**, **b** Proliferation of EJ and T24 cells transfected with cTFRC vector assessed using ^3^H-TdR incorporation at the indicated days. **c** Determination of cell invasive potential of EJ and T24 cells transfected with cTFRC vector by transwell assay. **d**, **e** Proliferation of EJ and T24 cells transfected with shCtrl, shcTFRC-1 or shcTFRC-1 + oe-cTFRC. **f**, **g** Transwell assays were used to evaluate the invasion in EJ and T24 cells after transfection with shCtrl, shcTFRC-1 or shcTFRC-1 + oe-cTFRC. Data are the means ± SD of three independent experiments. **P < 0.01

Tumor cell proliferation is a major representative indicator of malignant phenotypes. We noted that cTFRC was significantly up-regulated in bladder cancer compared with matched normal tissues (Fig. 1a), which prompted us to investigate the function of cTFRC in regulating BC cell proliferation. Subsequent cell proliferation assays showed that down-regulation of cTFRC significantly suppressed cell growth in a variety of BC cell types (Fig. 3a-c). However, knockdown of cTFRC had no effect on normal bladder epithelial cell HCV29 proliferation (Fig. 3d). To confirm these findings in the in vivo model, EJ or T24 cell lines were implanted subcutaneously into the pectoral region of nude mice (12 mice for the shCtrl group and 12 mice for the shcTFRC group). The volumes of the tumors were monitored once 5 days and consecutively for 5 weeks. As expected, silencing cTFRC markedly reduced the growth of the tumors in vivo (Fig. 3e). Furthermore, we examined the effects of overexpressing cTFRC on BC cell proliferation and invasion, and we found that cTFRC could promote BC cell proliferation and invasion (Fig. 4a-c). Finally, through the rescue experiment, we found that overexpression of cTFRC can restore the function of cell proliferation and invasion by cTFRC knockdown (Fig. 4d-g). Collectively, these results demonstrated that cTFRC may be capable of modulating the progression of BC.

### TFRC mediates cTFRC promoted BC progression

**Fig. 5:**
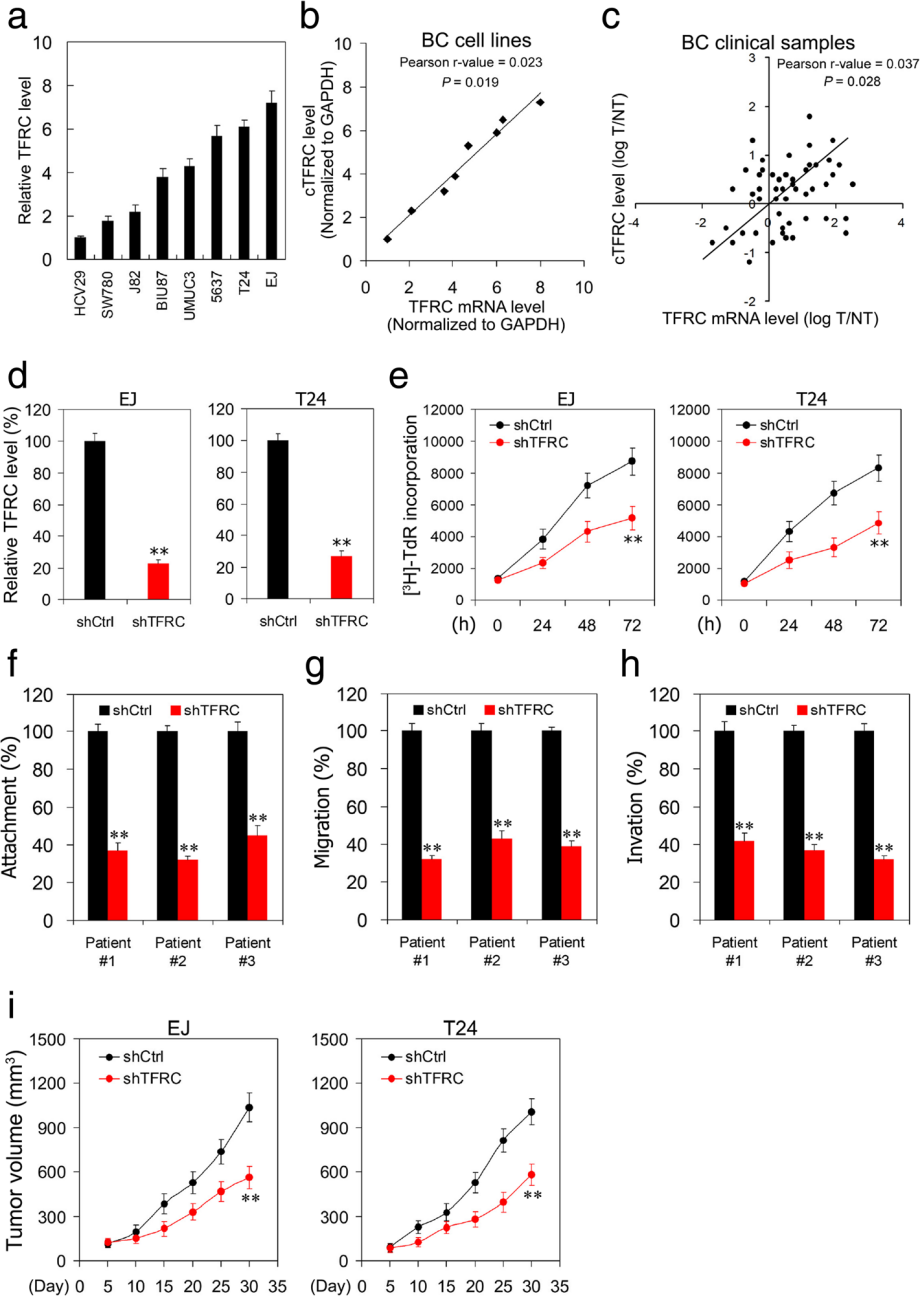
TFRC induces bladder cancer cell proliferation, migration and invasion. **a** qPCR analysis of TFRC expression in different BC cell lines. **b**, **c** cTFRC level correlated with TFRC mRNA expression in BC cell lines (**b**) and BC clinical samples (**c**). **d** TFRC was silenced in EJ and T24 BC cell lines by shRNAs and the TFRC-silenced stable cell lines were established. **e** Proliferation of EJ and T24 cells transfected with the above TFRC shRNA assessed using ^3^H-TdR incorporation at the indicated days. **f-h** TFRC depletion causes a diminished attachment (**f**), migration (**g**) and invasion (**h**) capacity in three BC patients’ primary cells. **i** TFRC-silenced EJ and T24 cells were subcutaneously injected into BALB/c nude mice for observation of tumor growth. Results are shown as mean ± SD. *n* = 12 for each group. Data are shown as mean ± SD. **P < 0.01 by two-tailed Student’s t test. Data represent at least three independent experiments

After determining the role of cTFRC in modulating BC cell proliferation and invasion, we then investigated the role of cTFRC in the regulation of BC progression by its host gene, TFRC. We first examined the TFRC mRNA levels in a variety of bladder cancer cell lines (Fig. 5a), and we found that TFRC mRNA expression was correlated with cTFRC expression in BC cell lines (Fig. 5b). Simultaneously, we detected the mRNA levels of TFRC and cTFRC level in BC clinical samples, and found that cTFRC expression was correlated with TFRC in clinical samples (Fig. 5c). Because cTFRC expression was significantly up-regulated in BC tissues and cTFRC expression was correlated with TFRC expression in BC cell line, we then analyzed TFRC expression in TCGA BC RNA-seq data. The results showed that TFRC mRNA expression level was obviously up-regulated in BC tissues group as compared with adjacent normal tissues group (Additional file 1: Figure S1A, B). To explore the clinical relevance of TFRC in TCGA BC patients, the correlation between TFRC expression levels and tumor grade, T stage and tumor lymphatic metastasis were evaluated. The results showed that TFRC was positively correlated with the tumor grade at mRNA levels, also T stage and tumor lymphatic metastasis (Additional file 1: Figure S1C-E). Furthermore, we also found that TFRC expression in BC tissues was significantly correlated with poor prognosis of BC patients, shown as a Kaplan-Meier survival curve using the median value of TFRC as the cut-off (Additional file 1: Figure S1F). Together, these data suggested that increased TFRC expression in BC was correlated with poor prognosis in TCGA BC patients.

To investigate the biological function of TFRC, we stably transfected EJ and T24 BC cells with shTFRC. The expression of TFRC was confirmed by qPCR (Fig. 5d). By using (^3^H) Thymidine incorporation, we determined that knockdown of cTFRC greatly impaired the cell proliferation ability of EJ and T24 cells (Fig. 5e). Consistently, TFRC silencing in 3 BC primary tumor cells also impaired cell attachment, migration and invasion (Fig. 5f-h). More importantly, we conducted xenograft tumor growth on TFRC knockdown of EJ and T24 BC cell lines. We noticed that underexpression of TFRC suppressed the growth of EJ and T24 BC cell xenografts within 30 days (Fig. 5i). Together, these data from BC specimens and BC cell lines showed that TFRC expression was correlated with the invasive and metastatic properties of BC.

### cTFRC and TFRC mediates TGFβ-induced EMT in BC cells

**Fig. 6:**
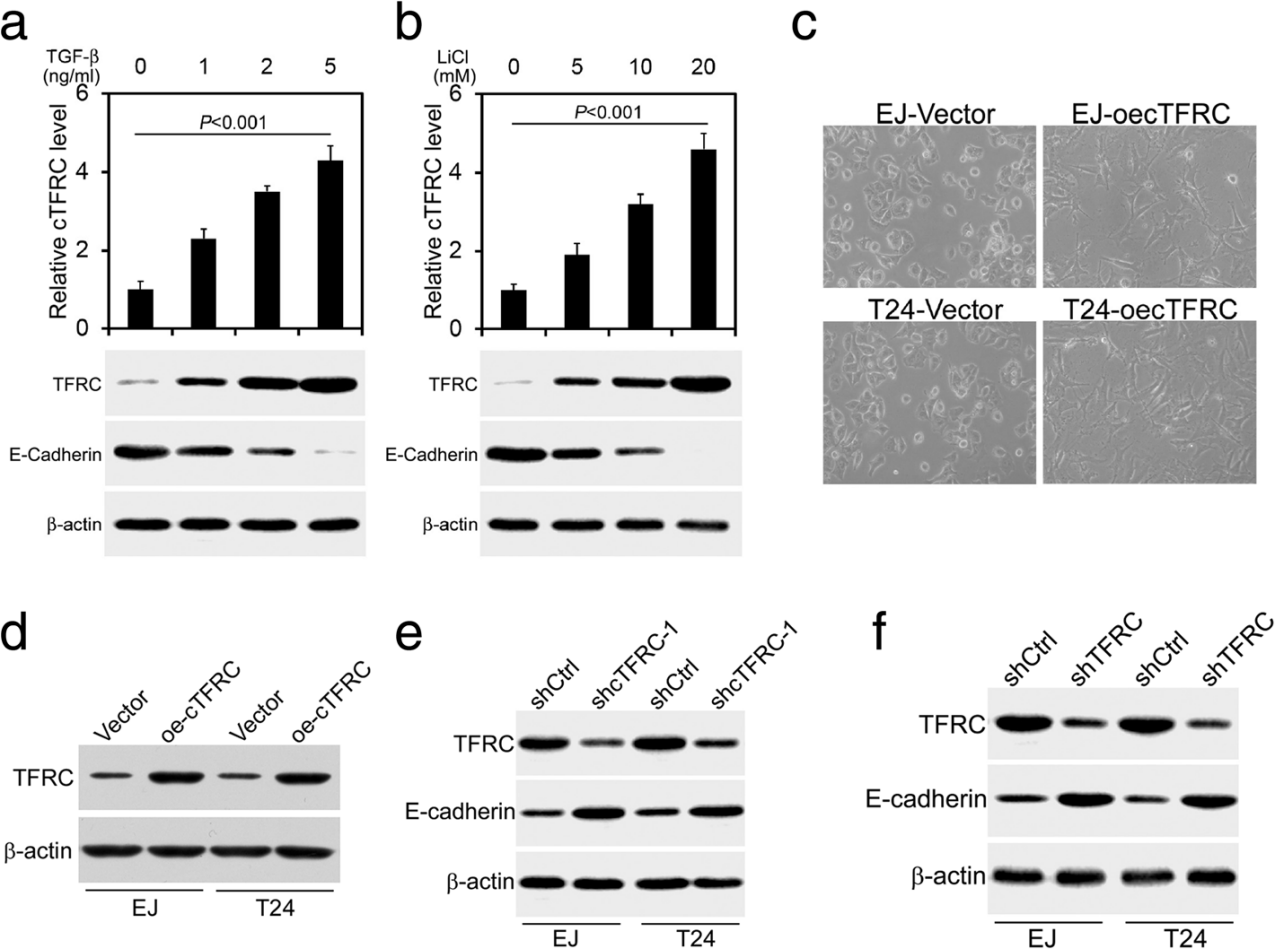
cTFRC mediates TGFβ-induced EMT. **a-b** qRT-PCR or western blot analysis of cTRFC, TFRC and E-cadherin levels: the protein expression of E-cadherin was reduced, while the expressions of cTFRC and TFRC were increased in EJ cells treated with TGF-β or LiCl, in comparison with those at 0 mM (control). β-actin was used as an internal control. **c** Phase contrast images of EJ or T24 cells 72 h after cTFRC-Vector transfection, showing an EMT-like morphology in cTFRC treated cells. **d** Western blot analysis of TFRC expression in overexpression cTFRC EJ or T24 cells. **e** TFRC and E-cadherin expression in shcTFRC and shCtrl-treated EJ or T24 groups. **f** TFRC and E-cadherin expression in shTFRC and shCtrl-treated EJ or T24 groups. β-actin was used as an internal control

EMT is a central mechanism contributing to the invasion and metastasis of various cancers [17]. To understand whether cTFRC can mediate EMT and invasion and motility of BC cells, we examined the epithelial marker E-cadherin and phenotypic changes of BC cells. The profibrotic factor transforming growth factor β (TGF-β) plays a crucial role in driving EMT progression [18]. Thus, to elucidate whether cTFRC and TFRC can mediate TGF-β-induced EMT, we treated EJ cells with vehicle or TGF-β (1, 2, 5 ng/ml) for 24 h to induce EMT. RT-qPCR and western blot analysis showed that cTFRC and TFRC expression were induced by TGF-β. The feature of EMT occurrence is that the epithelial marker E-cadherin was down-regulated (Fig. 6a). Meanwhile, we observed that cTFRC and TFRC had the same changes when stimulated with Wnt pathway inducer LiCl (Fig. 6b). Moreover, we examined the effects of cTFRC on morphological changes in BC cells. EJ or T24 cells overexpression cTFRC displayed typical EMT morphological changes, as demonstrated by phenotypic transformation from the endothelial cobblestone shape to fibroblastic spindle-shaped morphology (Fig. 6c). Western blot analysis showed that overexpression cTFRC increased TFRC expression in BC cell lines (Fig. 6d). Knocking down of cTFRC decreased TFRC expression while that of E-cadherin was significantly up-regulated compared to shCtrl cells (Fig. 6e). Moreover, E-cadherin levels were also significantly up-regulated when knocking out TFRC expression in EJ and T24 cells (Fig. 6f). Thus, these results indicated that cTFRC and TFRC expression mediated BC cell EMT.

### cTFRC serves as a sponge for the miR-107 and suppresses miR-107 activity

**Fig. 7:**
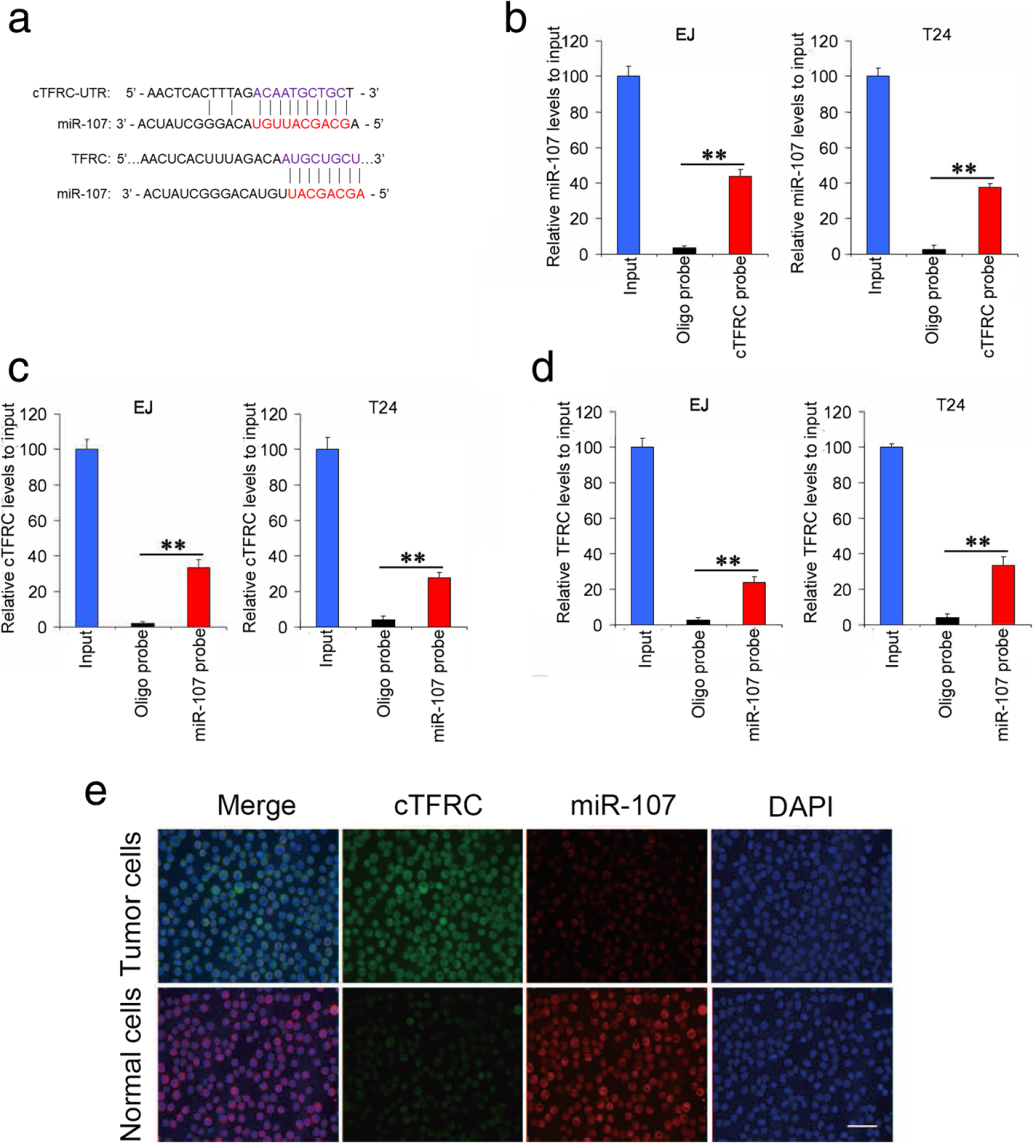
cTFRC targets miR-107 and inhibits its activity. **a** Binding sites of miR-107 in 3’UTR of cTFRC and TFRC. **b** EJ or T24 cells lysates were subject to RNA pull-down assay and tested by RT–PCR. Relative level of miR-107 was normalized to input. miR-107 can be pulled down by cTFRC probe. **c** Lysates prepared from EJ or T24 cells were subject to RNA pull-down assay and tested by RT–PCR. Relative expression of cTFRC was normalized to input. cTFRC can be pulled down by miR-107 probe. **d** miR-107 colocalized with cTFRC in BC primary tumor cells and adjacent normal cells were detected by FISH. Data are from three independent experiments. Magnification, 400X. **e** TFRC can be pulled down by miR-107 probe in EJ or T24 cells. Data are shown as mean ± SD. **P < 0.01 by two-tailed Student’s t test. Data represent at least three independent experiments

It has been reported that circRNA functions as “miRNA sponge” in cancer cells [19]. To address whether cTFRC could sponge miRNAs in bladder cancer cells the circRNA/microRNA interaction was predicted with Arraystar’s home-made miRNA target prediction software based on TargetScan & miRanda [20, 21]. According to the prediction, cTFRC possessed a complementary sequence to miR-107 seed region (Fig. 7a). Then to validate whether endogenous cTFRC serves as a binding platform for miR-107, we performed RNA pulldown assay in EJ and T24 cells. The endogenous miR-107 binding to cTFRC was specifically enriched by qPCR analysis (Fig. 7b), and the endogenous cTFRC pull-down by miR-107 was also significantly enriched in BC cells (Fig. 7c). Furthermore, by fluorescence in situ hybridization (FISH) analysis in BC primary tumor cells and matched normal tissues cells, we found that cTFRC was colocalized with miR-107 in the cytoplasm and this colocalization was decreased in tumor as compared to that in matched normal tissues cells (Fig. 7d). We next used the miRBase miRNA target prediction tool to find miR-107 that could bind to TFRC (Fig. 7a). The endogenous TFRC pull-down by miR-107 was also significantly enriched (Fig. 7e). All these experiments proved that cTFRC may function as a sponge for miR-107.

### cTFRC promotes the progression of BC through the miR-107-TFRC pathway

**Fig. 8:**
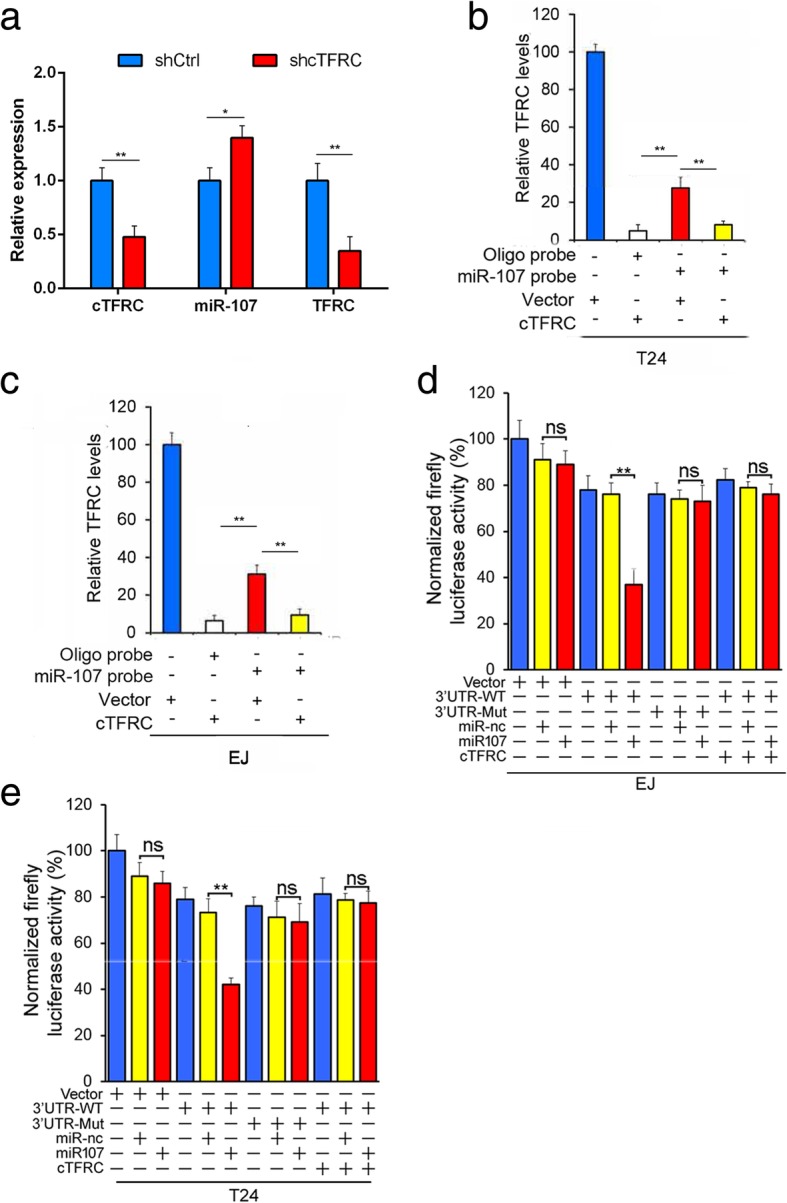
cTFRC specifically rescue the suppressive effects of miR-107 on TFRC expression. **a** qPCR analysis of cTFRC, miR-107 and TFRC expression in xenograft tumor from BC cell transfected with shcTFRC-1 or shCtrl in nude mice. **b**, **c** miR-107 RNA pull-down assay and qPCR analysis of TFRC expression in EJ or T24 cells lysates transfected with cTFRC or vector. **d**, **e** Luciferase reporter assay showed the luciferase intensity of EJ and T24 cells transfected with TFRC (wild/mutant) and miR-107 (nc/miR-107) or co-transfected with cTFRC and miR-107 (nc/miR-107)

In order to further confirm that cTFRC suppresses BC progression by sponge activity of miR-107 and consequently up-regulating TFRC, qPCR analysis showed that down-regulated cTFRC induce the expression of miR-107, while TFRC expression decreased in BC xenograft tissues (Fig. 8a). We next examined the miR-107 expression in different bladder cancer cell lines (Additional file 1: Figure S2A), also we found that miR-107 expression was negatively correlated with cTFRC expression in BC cell lines and BC clinical samples (Additional file 1: Figure S2B, C). More important, miR-107 mimc significantly blocked the BC cells invasion and proliferation activity (Additional file 1: Figure S2D-F). These data convincingly demonstrate that miR-107 promotes BC progression. Thus, we hypothesized that cTFRC acts as a sponge of miR-107 to eliminate the TFRC oncogenic effect through cTFRC/miR-107/TFRC axis. To test this hypothesis, we performed RNA-pulldown assay and found that the endogenous TFRC pull-down by miR-107 was significantly reduced if cTFRC existed (Fig. 8b and Fig. 8c). To further determine if cTFRC is a sponge of miR-107 to TFRC we used a luciferase activity assay to evaluate whether TFRC is a direct target of miR-107. The fragments of TFRC 3’-UTR containing the miR-107 binding sites were subcloned into the pmirGLO dual-luciferase reporter vector and then co-transfected with miR-107 mimic or miRNA-negative control. The results showed a significant reduction of luciferase activity in both EJ and T24 cells with miR-107 mimic transfection, compared with the negative control. In addition, transfection with cTFRC or mutation of the predicted-binding site of miR-107 on the TFRC 3’-UTR can rescue luciferase activity (Fig. 8d and Fig. 8e). Taken together, these findings demonstrate that cTFRC protected TFRC by sponging out miR-107 in BC cells. These data convincingly demonstrate that cTFRC promote BC progression as a sponge of miR-107 to eliminate the TFRC oncogenic effect through cTFRC/miR-107/TFRC axis.

**Fig. 9:**
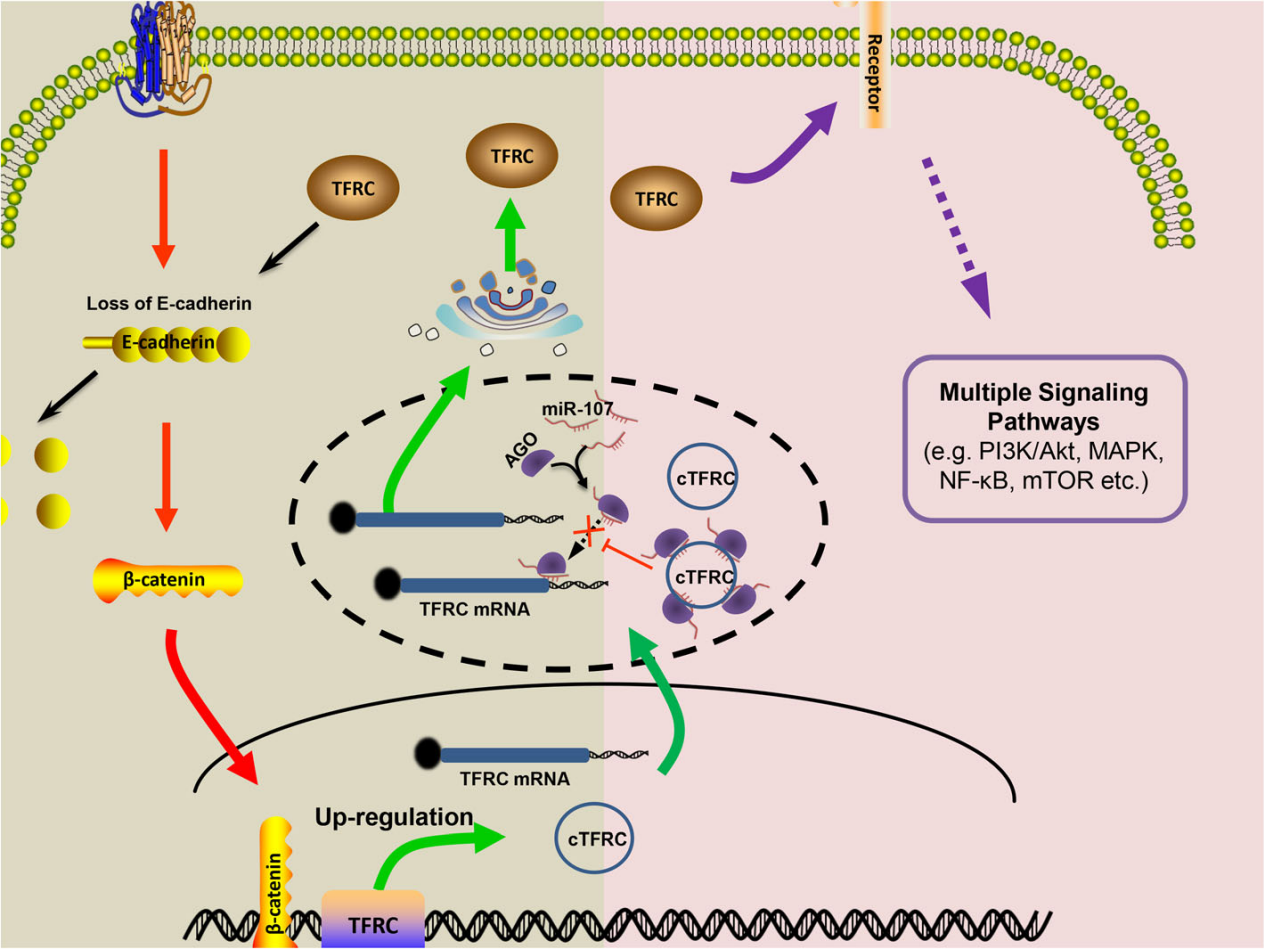
The schematic diagram shows the mechanism underlying cTFRC as a ceRNA for miR-107. Activation of the EMT signaling pathway in bladder cancer patients promotes transcriptional activation of TFRC, and expression of TFRC and cTFRC is increased in bladder cancer cells. On the one hand, TFRC can promote the degradation of E-cadherin, forming positive feedback to promote EMT, and ultimately promote cell invasion and migration. On the other hand, TFRC acts as a receptor to initiate multiple signaling pathways that promote downstream cell proliferation. In terms of mechanism, as shown by the dotted oval box, cTFRC upregulating the expression of TFRC by absorbs miRNA-107 targeting TFRC, thereby promoting epithellal-mesenchymal transition, cell proliferation and cell invasion in bladder carcinoma

Collectively, these findings provide first lines of evidences that cTFRC regulates TFRC expression through miR-107 contribute to the progression and EMT of BC (Fig. 9).

## Discussion

Numerous studies have shown that the expression profiles of non-coding RNAs (including lncRNAs and miRNAs) are abnormal in many types of cancers, and many of them focus on their epigenetic regulation in the development of cancer [22]. Recent studies have reported that many miRNAs and several lncRNAs may play a regulatory role in the development of BC [23, 24]. However, it is unclear whether circRNA plays a role in BC. In the past few years, the presence of circRNA has occasionally been recognized with covalent linkages in animal cells. Previously it was thought to be rare and even considered as transcriptional noise and artifacts [25]. However, circRNAs have recently been identified to be abundant stable ncRNAs by high-throughput sequencing and bioinformatics analysis [26]. It has been reported that circRNA is dysregulated in different cancer types such as colorectal cancer [27], liver cancer [19], esophageal squamous cell carcinoma [28], basal cell carcinoma [29] and laryngeal cancer [30]. These differentially expressed circRNAs may have some potential functions in the regulation of gene expression and have been widely accepted. CircRNA microarray results provide useful information for screening differentially expressed circRNAs and help us to select candidate circRNAs for further study.

Here, we identified a novel circular RNA through circRNA microarray analysis termed cTFRC that was significantly upregulated in human BC and correlated with clinical stage. Functionally, we found that down-regulation of cTFRC could inhibit cell invasion and proliferation, reduce EMT as well as facilitate tumor growth in vivo, whereas upregulation of cTFRC exerted an inductive role in EMT. Mechanistically, cTFRC could function as a ceRNA through harboring miR-107 to abolish the suppressive effect on the target gene TFRC in bladder cancer progression. Thus, our data suggest that cTFRC could play an important role in the pathogenesis and development of BC.

The role of circRNA in carcinogenesis and cancer progression has not been elucidated clearly. CircRNA may regulate the expression of oncogene or tumor suppressor gene through different targets, depending on the cancer or even at different stages. Together with miRNA and their target gene, the circRNA-miRNA-mRNA axis may serve as a wide network of gene expression regulators, and its deregulation may lead to disease progression, including cancer development. For example, circRNA SRY was reported to be a tumor-associated molecule in colorectal cancer and ovarian cancer by absorbing miR-138 [7]. It has been reported that circHIPK3 promotes proliferation of human liver cancer HuH-7 cells, human colon cancer HCT-116 cells, and human cervical cancer HeLa cells via sponging multiple miRNAs [31]. This assumption may lead to some interesting future work to elucidate the regulatory networks of non-coding RNAs and coding genes in cancer biology.

Although we have made encouraging progress in understanding the molecular mechanisms of BC, the prognosis of patients with advanced bladder cancer remains unfavorable. Therefore, it is of great significance to reveal the underlying mechanism of bladder cancer metastasis. Specifically, EMT is a powerful paradigm for studying the genetic progression of advanced stage solid tumors [32]. In addition, as most human malignancies originate from epithelial tissues, the investigation on EMT is not only beneficial to bladder cancer, but also to other solid tumors [33]. In the present study, we explored cTFRC on the EMT process of bladder cancer cell by regulating TFRC expression. Underexpression of cTFRC induced EMT process marker E-cadherin expression and suppression TFRC expression. Besides, overexpression of cTFRC resulted in morphological alteration and EJ-oecTFRC or T24-oecTFRC cells displayed elongated mesenchymal-like characteristics, indicating that these cells were undergoing epithelial to mesenchymal transition. Changes in morphology of EJ or T24 cells might occur after cTFRC regulates TFRC expression.

TFRC plays a pivotal role in iron cellular uptake, and cellular iron deficiency inhibits cell growth and leads to cell death [34]. In malignant tissues, TFRC expression is more than in their normal tissues, as cancer cells require large amounts of iron to maintain their high rate of cell proliferation [35]. Therefore, TFRCs are attractive targets for immunotherapy and cytotoxic delivery agents because of their increased expression on malignant cells as compared to normal cells. Recently, many reports found that TFRC is involved in tumor progression [36]. However, TFRC has not been reported on bladder cancer progression. In this study, we found that TFRC is overexpressed in bladder cancer and correlated with poor prognosis of BC patient. More interesting is that cTFRC expression is correlated with TFRC both in tumor cell lines and tumor tissues. Also, we found that cTFRC was overexpressed in bladder cancer and cTFRC up-regulated TFRC expression in bladder cancer as a “miRNA sponge”. Thus, this may explain the high expression of TFRC in BC patients, but little is known about cTFRC highly expressed in bladder cancer. Therefore, the regulation of cTFRC expression in BC also requires further exploration.

## Conclusion

We show that cTFRC is up-regulated in human bladder cancer, and it can efficiently sponge miR-107 to induce TFRC expression, as well as demonstrate that under-expression of cTFRC can effectively inhibit proliferative and invasive ability of bladder cancer cells through targeting miR-107/TFRC axis. The ceRNA network and pathway might be a new therapeutic target for the treatment of BC.

## Additional file

Additional file 1: **Figure S1.** TFRC expression is up-regulated and positively associated with advanced tumor grade of patients with BC. (A, B) TFRC mRNA expression in BC and their adjacent tissues, the data were obtained from TCGA BC data set (nonparametric Mann–Whitney test, **P < 0.01). (C) The high expression levels of TFRC in BC patients with high grade. (D) Advanced T stage is associated with higher TFRC levels. (E) The expression of TFRC higher in patients with lymphatic metastasis. (F) Prognostic significance of TFRC expression for TCGA BC patients was performed with TFRC values by using the median value as the cutoff. **Figure S2.** The expression correlation between miR-107 and cTFRC (A) qPCR analysis of miR-107 expression in different BC cell lines. (B, C) miR-107 level negatively correlated with cTFRC mRNA expression in BC cell lines (B) and BC clinical samples (C). (D) Determination of cell invasive potential of EJ and T24 cells transfected with miR-107 mimic by transwell assay. (B) Proliferation of EJ and T24 cells transfected with miR-107 mimic assessed using 3H-TdR incorporation at the indicated days. Data are shown as mean ± SD. **P < 0.01 by two-tailed Student’s t test. Data represent at least three independent experiments. **Table S1.** Primers for qRT-PCR analysis. **Table S2.** The sequences of the effective shRNAs. **Table S3.** The probes for fluorescence in situ hybridization. (PDF 787 kb)

## Abbreviations

BC: Bladder cancer
ceRNA: Competitive endogenous RNA
circRNA: Circular RNA
FISH: Fluorescence in situ hybridization
GAPDH: Glyceraldehyde 3-phosphate dehydrogenase
MIBC: Muscle invasive bladder cancer
miRNAs: microRNAs
MREs: miRNA response elements
NMIBC: Non-muscle invasive bladder cancer
qPCR: Quantitative RT-PCR
TFRC: Transferrin receptor
TGF-β: Transforming growth factor β
