# Supplementary material for: Circular RNA cTFRC acts as the sponge of MicroRNA-107 to promote bladder carcinoma progression
Source: Mol Cancer. 2019 Feb 19;18:27. doi: 10.1186/s12943-019-0951-0 (PMC6379985; doi:10.1186/s12943-019-0951-0)
Supplement: Supplementary file 1 — Figure S1. TFRC expression is up-regulated and positively associated with advanced tumor grade of patients with BC. (A, B) TFRC mRNA expression in BC and their adjacent tissues, the data were obtained from TCGA BC data set (nonparametric Mann–Whitney test, **P < 0.01). (C) The high expression levels of TFRC in BC patients with high grade. (D) Advanced T stage is associated with higher TFRC levels. (E) The expression of TFRC higher in patients with lymphatic metastasis. (F) Prognostic significance of TFRC expression for TCGA BC patients was performed with TFRC values by using the median value as the cutoff. Figure S2. The expression correlation between miR-107 and cTFRC (A) qPCR analysis of miR-107 expression in different BC cell lines. (B, C) miR-107 level negatively correlated with cTFRC mRNA expression in BC cell lines (B) and BC clinical samples (C). (D) Determination of cell invasive potential of EJ and T24 cells transfected with miR-107 mimic by transwell assay. (B) Proliferation of EJ and T24 cells transfected with miR-107 mimic assessed using 3H-TdR incorporation at the indicated days. Data are shown as mean ± SD. **P < 0.01 by two-tailed Student’s t test. Data represent at least three independent experiments. Table S1. Primers for qRT-PCR analysis. Table S2. The sequences of the effective shRNAs. Table S3. The probes for fluorescence in situ hybridization. (PDF 787 kb) [file 12943_2019_951_MOESM1_ESM.pdf]

Supplemental Figure Legends

Figure S1. TFRC expression is up-regulated and positively associated with advanced tumor grade of patients with BC. (A, B) TFRC mRNA expression in BC and their adjacent tissues, the data were obtained from TCGA BC data set (nonparametric Mann–Whitney test, $^{**}P < 0.01$). (C) The high expression levels of TFRC in BC patients with high grade. (D) Advanced T stage is associated with higher TFRC levels. (E) The expression of TFRC higher in patients with lymphatic metastasis. (F) Prognostic significance of TFRC expression for TCGA BC patients was performed with TFRC values by using the median value as the cutoff.

Figure S2. The expression correlation between miR-107 and cTFRC (A) qPCR analysis of miR-107 expression in different BC cell lines. (B, C) miR-107 level negatively correlated with cTFRC mRNA expression in BC cell lines (B) and BC clinical samples (C). (D) Determination of cell invasive potential of EJ and T24 cells transfected with miR-107 mimic by transwell assay. (B) Proliferation of EJ and T24 cells transfected with miR-107 mimic assessed using 3H-TdR incorporation at the indicated days. Data are shown as mean ± SD. $^{**}P < 0.01$ by two-tailed Student's t test. Data represent at least three independent experiments.

Figure S1

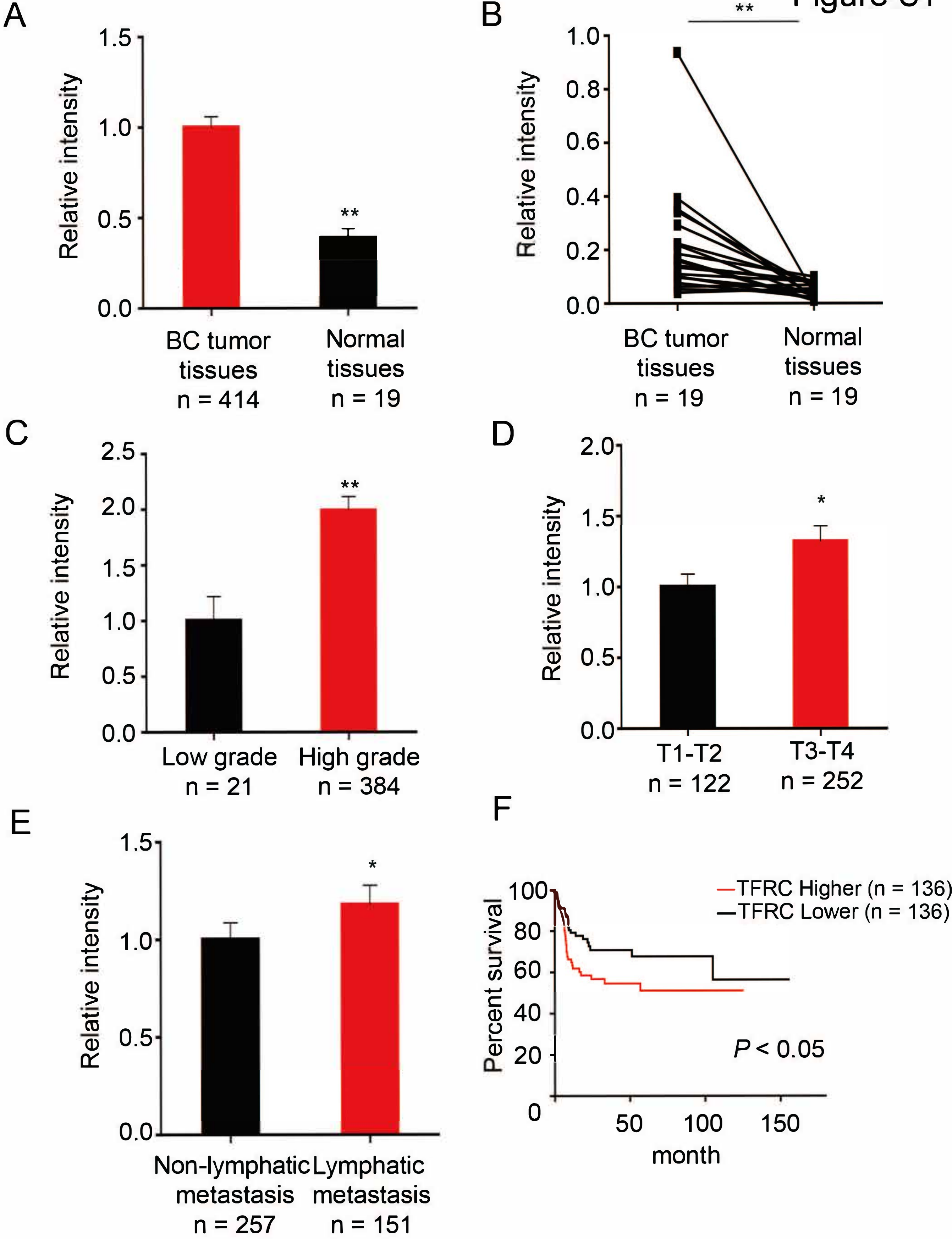
A
Relative intensity
1.5
1.0
0.5
0.0
**
BC tumor tissues n = 414
Normal tissues n = 19
B
**
Relative intensity
1.0
0.8
0.6
0.4
0.2
0.0
BC tumor tissues n = 19
Normal tissues n = 19
C
Relative intensity
2.5
2.0
1.5
1.0
0.5
0.0
**
Low grade n = 21
High grade n = 384
D
Relative intensity
2.0
1.5
1.0
0.5
0.0
*
T1-T2 n = 122
T3-T4 n = 252
E
Relative intensity
1.5
1.0
0.5
0.0
*
Non-lymphatic metastasis n = 257
Lymphatic metastasis n = 151
F
TFRC Higher (n = 136)
TFRC Lower (n = 136)
Percent survival
100
80
60
40
20
0
P < 0.05
50
100
150
month

Figure S2

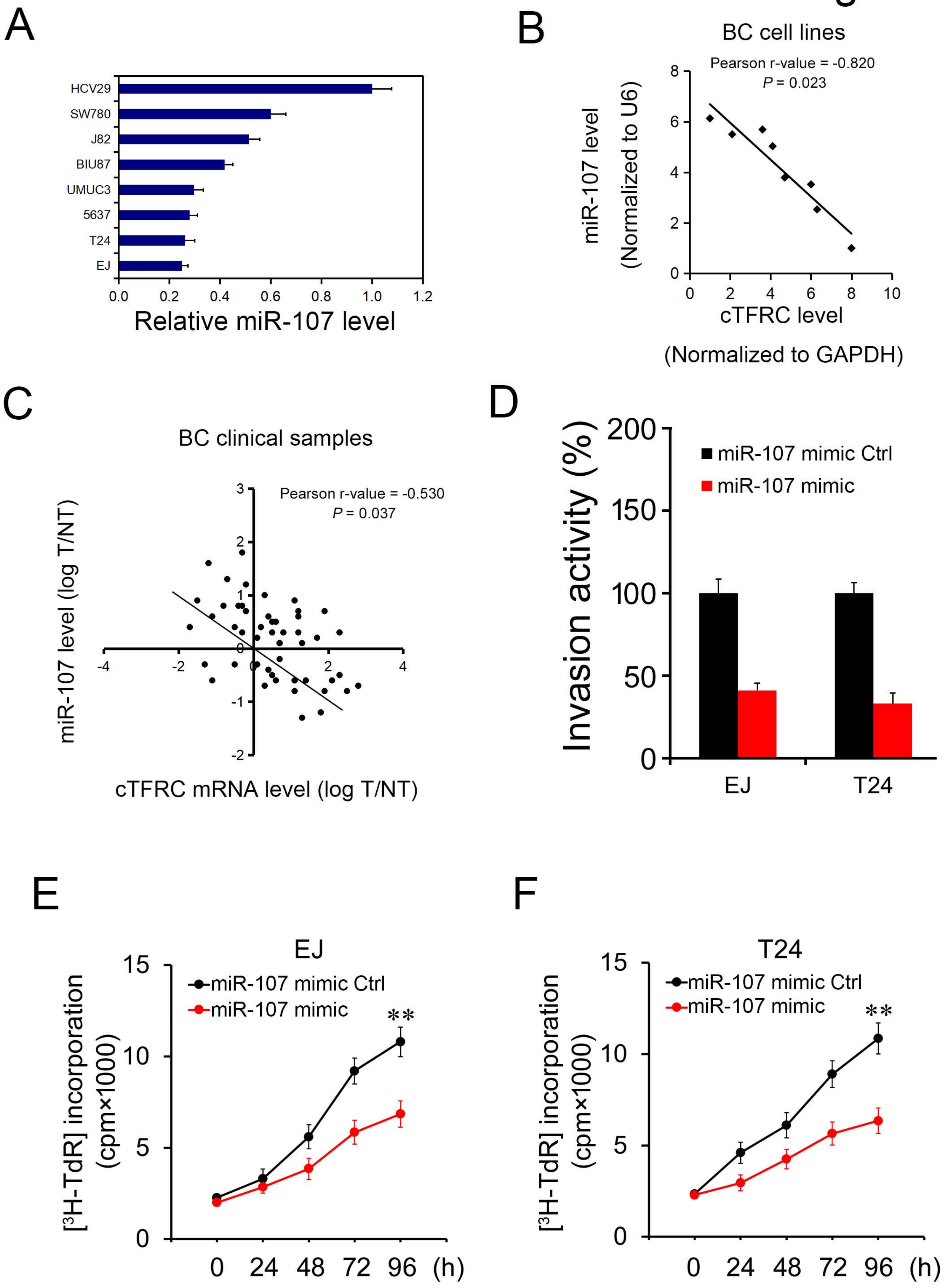
A
HCV29
SW780
J82
BIU87
UMUC3
5637
T24
EJ
0.0 0.2 0.4 0.6 0.8 1.0 1.2
Relative miR-107 level
B
BC cell lines
Pearson r-value = -0.820
P = 0.023
miR-107 level
(Normalized to U6)
cTFRC level
(Normalized to GAPDH)
C
BC clinical samples
Pearson r-value = -0.530
P = 0.037
miR-107 level (log T/NT)
cTFRC mRNA level (log T/NT)
D
Invasion activity (%)
miR-107 mimic Ctrl
miR-107 mimic
EJ
T24
E
EJ
[3H-TdR] incorporation
(cpm×1000)
miR-107 mimic Ctrl
miR-107 mimic
**
0 24 48 72 96 (h)
F
T24
[3H-TdR] incorporation
(cpm×1000)
miR-107 mimic Ctrl
miR-107 mimic
**
0 24 48 72 96 (h)

Table S1. Primers for qRT-PCR analysis

Name	Sequences (5'—3')
qRT-PCR primers*	
cTFRC	F: TGCTGTCCAGCAGCCATAGG; R: TCATTCTGAACTGCCACACAGA
TFRC	F: ACTTGCCCAGATGTTCTCAG; R: GTATCCCTCTAGCCATTCAGTG
U6	F: CGAGCACAGAATCGCTTCA; R: CTCGCTTCGGCAGCACATAT
GAPDH	F: AAGGTGAAGGTCGGAGTCAA; R: GGAAGATGGTGATGGGATTT
ATCB	F: TCCCAACAGCTT; R: ATGACGTCTGGGCCTGTCTA
miR-107	F: CAGCAGCATTGTACAGGGCTATCA; R: GGTCCAGTTTTTTTTTTTTTTTGAC

* F, forward; R, reverse.

Table S2. The sequences of the effective shRNAs

Name	Sequences (5'—3')
shcTFRC-1	TTGCGAGGTACCAGCAACT
shcTFRC-2	CTTTCTGTTTTTGCGAGGT
shcTFRC-3	AACTTCAAGGTTTCTGCCA

Table S3. The probes for fluorescence in situ hybridization

Name	Sequences (5'—3')
cTFRC probe	GGA AGG ATT ACA TGA CAT CTG ACC CAA AAC AAC CCC ACT GAC
miR-107 probe	TCT TTG GTT ATC TAG CTG TAT GA
